# Warfarin-associated fetal intracranial subdural hematoma: a case report

**DOI:** 10.1002/ccr3.75

**Published:** 2014-05-18

**Authors:** Kana Fujiwara, Shigeru Aoki, Kentaro Kurasawa, Mika Okuda, Tsuneo Takahashi, Fumiki Hirahara

**Affiliations:** 1 Perinatal Center for Maternity and Neonate, Yokohama City University Medical CenterYokohama, Japan; 2 Department of Obstetrics and Gynecology, Yokohama City University HospitalYokohama, Japan

**Keywords:** Anticoagulation, fetal intracranial hemorrhage, heparin, mechanical heart valve replacement, warfarin

## Abstract

**Key Clinical Message:**

We present a case in which to of fetal subdural hematoma developing despite that the maternal the prothrombin time by international normalized ratio (PT/INR) during pregnancy was within the normal range.

## Introduction

In pregnant women with mechanical heart valve replacement, anticoagulation is essential for the prevention of maternal thromboembolism. A study on the use of heparin alone throughout pregnancy showed that thromboembolic complications and maternal death occurred in 25% and 6.7% of subjects, respectively [1]. Because the preventive effect of heparin on thrombosis is uncertain, switching to oral administration of warfarin is necessary [2]. Because warfarin crosses the placenta, it can cause complications in both mothers and fetuses. However, oral administration of this drug at a dose below 5 mg/day is associated with a low frequency of complications and was reported to be relatively safe [3].

We present a case of warfarin-associated fetal subdural hematoma at 31 week of gestation.

## Case

**Figure 1:**
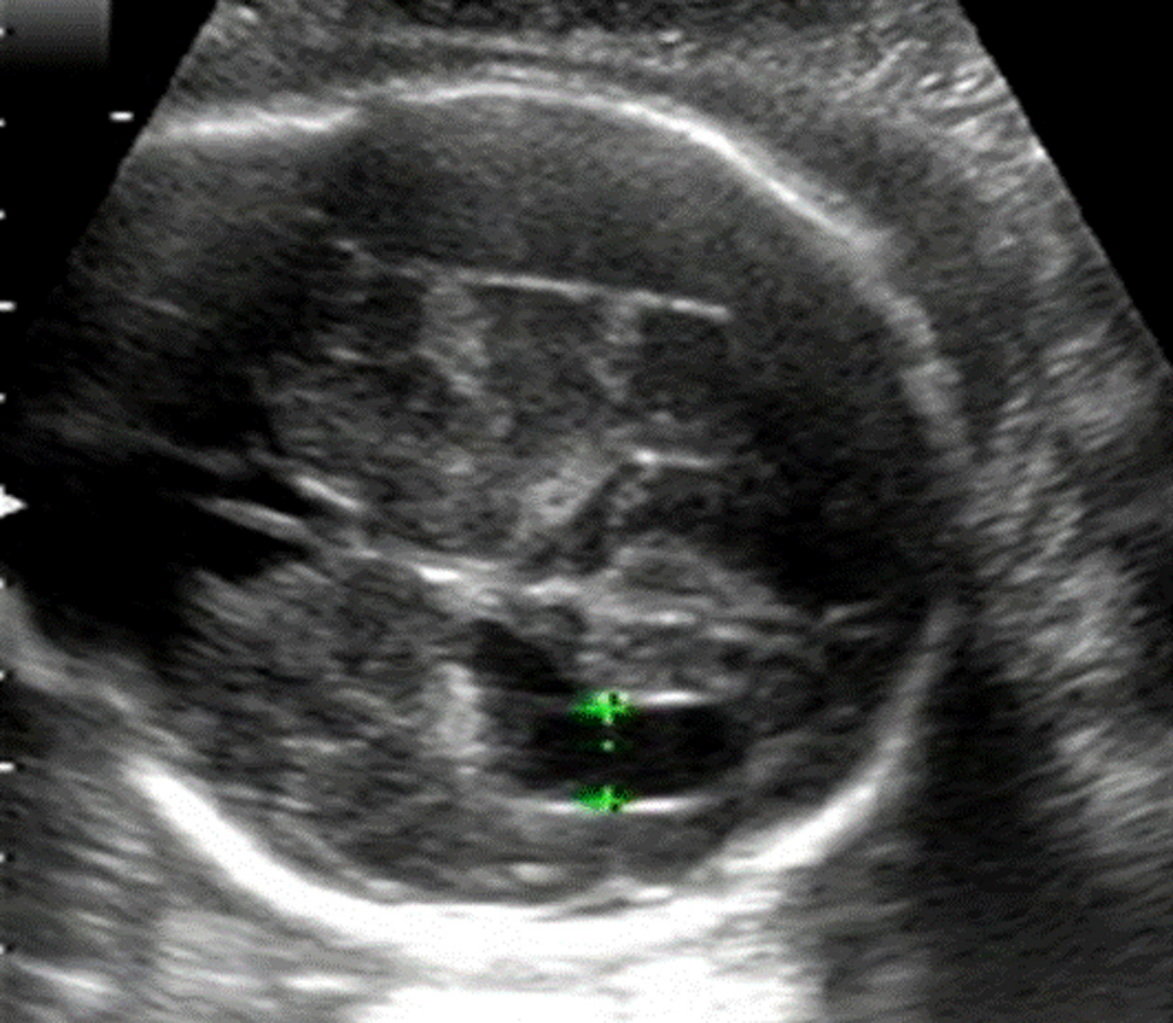
Head of the fetus at the 33 weeks of gestation. Transabdominal ultrasonography reveals enlargement of the left lateral ventricle and hematoma in the right subdural area.

**Figure 2:**
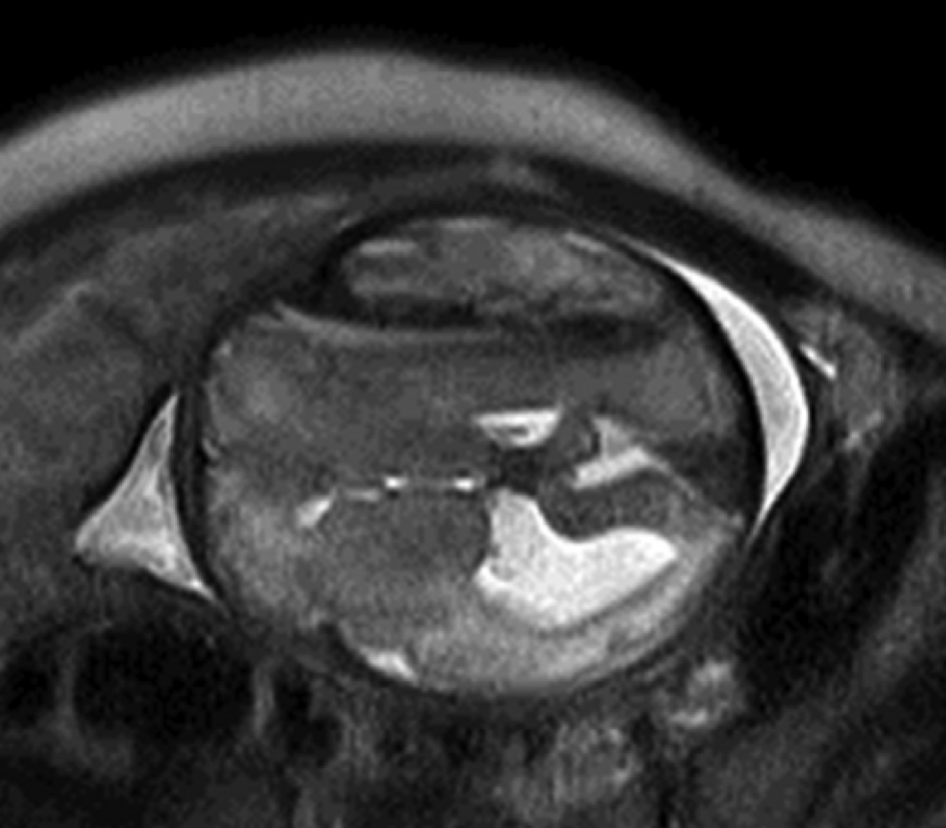
Head of the fetus in the mother's pelvis. The T2-weighted MRI images show hematoma in the bilateral subdural areas and enlargement of the left lateral ventricle due to compression.

A 27-year-old woman, gravida 2, para 1, had undergone aortic valve replacement (mechanical) for aortic regurgitation at the age of 10 years. Since then, she had been receiving anticoagulation with warfarin, 5 mg/day. During the previous pregnancy, she received a combination of warfarin and heparin for anticoagulation, and the pregnancy and delivery were uneventful for both mother and infant. As with the previous pregnancy, oral administration of warfarin was switched to continuous intravenous infusion of heparin in the fifth week of gestation. In the 12 weeks of gestation, oral administration of warfarin was resumed at a dose of 3 mg/day, and then, the dose was increased to 4.5 mg/day. The activated partial thromboplastin time (APTT) was maintained between 50 and 60 sec during heparin use, and prothrombin time by international normalized ratio (PT/INR) was maintained between 1.5 and 2.0 during oral administration of warfarin. On the fifth day of the 31 week of gestation, ultrasonography revealed mild enlargement of the left lateral ventricle of the fetus, measuring 1.0 cm, and it was found to have further enlarged to 1.5 cm on the fifth day of the 33 weeks of gestation (Fig.1). Magnetic resonance imaging (MRI) performed on the first day of the 35 week of gestation revealed bilateral intracranial subdural hematoma in the fetus, and the left ventricle was found to have enlarged due to compression by the hematoma (Fig.2). We determined that the fetal intracranial subdural hematoma had been caused by fetal intracranial hemorrhage associated with warfarin administration to the mother. On the same day, warfarin was discontinued and replaced with continuous intravenous infusion of heparin. Cardiotocography (CTG) showed that the fetal conditions were favorable and no anemia was observed based on the measurement of middle cerebral artery peak systolic velocity (MCA-PSV). On the second day of the 36 weeks of gestation, the mother underwent elective induction of delivery with oxytocin in response to her desire for a vaginal delivery. A male infant of 2648 g, with Apgar scores of 8 and 9 at 1 and 5 min, respectively, was delivered and umbilical arterial blood pH was 7.266. He had good muscle tone with no apparent paralysis of the limbs. Both respiration and circulation were well maintained. Because the infant had a hemoglobin level of 7.0 mg/dL, PT/INR of 4, and APTT of 105 sec, showing anemia and abnormal coagulation parameters, red cell concentrate and fresh-frozen plasma were transfused. At 3 days of age, he underwent cranial surgery with bilateral subdural hematoma removal. At present, 4 months after birth, he remains asymptomatic with no motor deficits or growth problems restriction.

## Discussion

The earlier reported causes of fetal subdural hematoma include changes in maternal blood pressure, epilepsy, abdominal trauma, placental abruption, treatment with or exposure to certain drugs (e.g., warfarin and cocaine), intrauterine infections, thrombocytopenia, coagulopathies, hydrops fetalis, and congenital vascular malformations [4]. In our case, the hematoma developed after resumption of warfarin treatment. Because there were no causes other than this drug, warfarin-associated fetal intracranial subdural hematoma was diagnosed. Warfarin can cause fetal intracranial hemorrhage even at a daily dose as low as 5 mg or less. It was suggested that findings of ventricular enlargement on intensive ultrasonography and fetal MRI are useful for early detection of fetal intracranial hemorrhage.

Although PT/INR was adequately maintained between 1.5 and 2.0 on oral warfarin at a dose of 4.5 mg/day for anticoagulation after mechanical heart valve replacement, fetal intracranial hemorrhage occurred. These patients are at a high risk of thromboembolism, and it is recommended that warfarin should be switched to heparin therapy during the 6–12th week of gestation for the concern of teratogenicity and 2–3 weeks before delivery for possible maternal and/or fetal hemorrhages during delivery. During other periods, the use of warfarin is recommended [2]. However, because warfarin can cross the placenta, there is concern over the impact of this drug on the fetus. The incidence of warfarin-associated fetal intracranial hemorrhage is very low, but a few cases have been reported [5–8]. In a study that Bian et al. [3] conducted on the relationship between warfarin dosage and fetal complications, neither stillbirth nor neonatal death occurred in 58 pregnant women whose PT/INR was maintained between 1.5 and 2.0 on a warfarin dosage of less than 5 mg/day, and the clinical courses were favorable in both mothers and fetuses. Based on this, Bian et al. reported that a warfarin dosage below 5 mg/day is relatively safe [9,10]. However, there are also reports of fetal intracranial hemorrhage occurring in gestations of women whose PT/INR was maintained at ∼2 on a warfarin dosage of less than 5 mg/day [11], as seen in our case. Thus, the safety of the fetus cannot be guaranteed by either a low warfarin dosage or adequately maintained PT/INR.

Ultrasound examination and identification of enlargement of the lateral ventricle by fetal MRI are considered useful for early identification of fetal intracranial hemorrhage. Fetal intracranial hemorrhage was suspected in the 31 weeks of gestation on ultrasonography, and a live infant was delivered in the 36 weeks of gestation. Our search of PubMed revealed six reported cases of fetal intracranial hemorrhage associated with warfarin. Only one of these infants was born alive, making our case to be the second. Fetuses with a diagnosis of intracranial hemorrhage have a very poor prognosis (Table1). We detected mild enlargement of the lateral ventricle in the fetus at 31 weeks of gestation, and further enlargement was observed 2 weeks later. It is not physiologically possible for fetal lateral ventricle enlargement to occur in the third trimester and asymmetric enlargement does not occur at any time during gestation. Therefore, we suspected fetal intracranial hemorrhage, which led to MRI diagnosis at the 35 weeks of gestation. In the other live-born infant, reported by Matsuda et al. [7], fetal intracranial hemorrhage was diagnosed by ultrasonography and MRI in the 31 weeks of gestation, and cesarean section was performed after corticosteroid administration. Our patient delivered a live infant and the neurological prognosis is favorable at present. However, when the lateral ventricle in the fetus showed progressive enlargement on the fifth day of the 33 weeks of gestation, we should possibly have performed MRI immediately and then delivered. Because all seven cases of fetal intracranial hemorrhage associated with warfarin, including the infant in our case, show enlargement of the ventricles, caution is necessary when interpreting ultrasound findings of enlarged ventricles during the use of warfarin. Moreover, when an enlarged ventricle is detected, fetal MRI should immediately be performed to make a definitive diagnosis [4].

**Table 1:** Warfarin-associated fetal intracranial hemorrhage in pregnant women with mechanical heart valves from six previously published cases and the current case

	Masamoto et al. [5]	Lee et al. [6]	Matsuda et al. [7]	Oswal and Agarwal [8]	Ville et al. [11]	Ville et al. [11]	Present case
Warfarin dose (mg/day)	3.5	6–7	NA	NA	3–6	5–6	3–4.5
Start of warfarin (weeks)	20	14	14	24	26	15	12
PT (INR)	2.7–2.9	2.2–3.0	NA	NA	1.7–2.5	0.7–1.9	1.5–2.0
GA at diagnosis (weeks)	23	23	31	24	32	29	31
Hemorrhage site	Subdural	Subdural	Subdural	Subdural	Intraventricular	Intraventricular	Subdural
Delivery (weeks)	23	25	31	24	36	29	36
Fetal outcome	Stillbirth	Neonatal death	Alive	Termination	Stillbirth	Stillbirth	Alive

Of the seven reports of warfarin-associated fetal cerebral hemorrhage to date, this is only the second to describe delivery of a live baby. GA, gestational age (weeks); PT/INR, prothrombin time/international normalized ratio; NA, not available.

In conclusion, even when PT/INR is adequately maintained on oral warfarin at a dose of less than 5 mg/day, fetal intracranial hemorrhage can occur. The course of our patient suggests that early detection of fetal intracranial hemorrhage requires intensive ultrasonography, and that fetal MRI is useful in cases with findings suggestive of intracranial hemorrhage. Although the use of warfarin is essential for reducing the risk of thromboembolism and maternal death in pregnant women with mechanical heart valve replacement, it is important to sufficiently counsel these women regarding the possible effects of this drug on the fetus.
